# Evaluation of the quality of life and risk of suicide

**DOI:** 10.6061/clinics/2016(03)03

**Published:** 2016-03

**Authors:** Verônica de Medeiros Alves, Leilane Camila Ferreira de Lima Francisco, Flaviane Maria Pereira Belo, Valfrido Leão de-Melo-Neto, Vinicius Gomes Barros, Antonio E Nardi

**Affiliations:** I Universidade Federal de Alagoas, Departamento de Enfermagem, Arapiraca/AL, Brasil; II Universidade Federal de Alagoas, Faculdade de Medicina, Maceió/AL, Brasil; III Universidade Federal do Rio de Janeiro, Instituto de Psiquiatria, Rio de Janeiro/RJ, Brasil

**Keywords:** Mental Disorders, Suicide Attempts, Suicidal Ideation, Quality of Life

## Abstract

**OBJECTIVE:**

To identify the socio-demographic profiles, suicidal ideation, the presence of mental disorders and the quality of life of patients using mental health services in Arapiraca, Alagoas, Brazil.

**METHOD:**

Interviews were conducted in family health units and the Psychosocial Attention Center. The sample included 202 mental disorder patients with a risk of suicide attempts, 207 mental disorder patients without a risk of suicide attempts and 196 controls. This study used an identification questionnaire, the abbreviated World Health Organization Quality of Life questionnaire, Beck‘s Suicidal Ideation Scale and the Mini International Neuropsychiatric Interview.

**RESULTS:**

Patients who had a mental disorder and a risk of suicide attempts tended to be single, had less education and lower family income, were not working and showed lower scores in quality of life domains; 73 of these patients had suicidal ideation in the previous week. Depressive disorders, manic episodes, hypomanic episodes, social phobias, obsessive compulsive disorder, post-traumatic stress disorder, psychotic syndromes and generalized anxiety disorder were more frequent and statistically significant for patients at risk for suicide attempts.

**CONCLUSION:**

The management of patients with a risk of suicide attempts must focus on individual patients because this risk is directly linked to changes in quality of life and the improvement of these patients' prognosis.

## INTRODUCTION

In 2014, the World Health Organization (WHO) 1 reported that almost 804,000 people commit suicide every year. According to the United Nations, 75% of these cases involve people from low- and middle-income countries. Brazil ranks eighth in the world for the number of suicides. In 2012, 11,821 deaths due to suicide were recorded in Brazil, including 9,198 men and 2,623 women. Between 2000 and 2012, the number of suicides increased by 10.4% 1.

Suicidal behavior encompasses a number of phenomena linked to suicide, of which the most relevant are the suicide itself (death) and suicide attempts 2. Suicide attempts have the same phenomenological characteristics of suicides and differ only in terms of the outcome, which is not fatal in the case of suicide attempts 2.

It is believed that suicide attempts occur at least ten times more often than suicides, but official records regarding suicide attempts are scarcer and less reliable than those regarding suicide 3. There are no worldwide records on suicide attempts. It may be impossible to accurately obtain these data because most of these attempts are never recorded in medical or legal contexts. It is estimated that the rates of suicide attempts may be 10 to 40 times higher than the rates of suicide 4. In addition, there may be at least ten times as many suicide attempts for each suicide and there may be four unrecorded suicide attempts for each attempt recorded 5.

Patients with mental disorders often complain of suicide attempts and/or suicidal ideation. Risk factors for suicide are social isolation, depression, schizophrenia, bipolar disorder, alcoholism, unemployment, extreme economic situations, difficult family relationships, a parental loss in childhood, a recent loss of a close friend or family member, organic diseases causing disability, chronic pain, malignant neoplasms and AIDS 3. In addition, a family history of suicide is considered an aggravating factor 6.

Due to the consequences that suicide attempts generally introduce in the lives of patients and their families, this study aims to identify the socio-demographic profiles, suicidal ideation, the presence of mental disorders and the quality of life of patients who are receiving mental health services and who are at risk of suicide in Arapiraca, Alagoas, Brazil.

## MATERIALS AND METHODS

This quantitative research was conducted at the Psychosocial Attention Center (PAC) Nise da Silveira and in family health units (FHU) located in Arapiraca, Alagoas, Brazil.

The sample consisted of 605 people: 202 mental disorder patients with a risk of suicide attempts (the MDRS group); 207 mental disorder patients without a risk of suicide attempts (the MDWS group); and 196 patients without mental disorders and without a risk of suicide attempts, who were the study's control group (the CO group).

The exclusion criteria were patients with mental retardation and people who were younger than 18 years of age. The Research Ethics Committee of the Federal University of Alagoas approved this study, and the subjects expressed their acquiescence through informed consent.

The following scales and questionnaires were used: an identification questionnaire, the abbreviated WHO Quality of Life questionnaire (WHOQOL), Beck's Suicidal Ideation Scale and the Mini International Neuropsychiatric Interview (MINI 5.0) 7.

The identification questionnaire provided an economic profile and the social and health conditions of the patients studied. The WHOQOL allowed for an evaluation of the patient's quality of life in four domains: the physical, the psychological, social relations and the environment 8. The Suicidal Ideation Scale was used to identify people who had had suicidal ideation in the previous week 9. The MINI 5.0 was used as a diagnostic interview. The control group consisted of patients who showed no mental disorder or suicide risk during the MINI.

The analysis was conducted using the statistical program SPSS 20. Pearson's correlation, x^2^ tests, and Student's t tests were used, with a significance value of *p*≤0.05.

## RESULTS

The MDWS group's average age was greater than the ages of the other groups (*p*<0.05). Most of the patients interviewed were female (Table 1).

Most patients in the MDRS group were single or divorced, whereas most of the patients in the MDWS group (*p*<0.05) and the CO group were married (*p*>0.05) (Table 1).

The level of education of the MDRS patients was lower than that of the MDWS group (*p*<0.05) and that of the CO group (*p*<0.05). A high level of education was found in the CO group (*p*<0.05), followed by the MDWS group (*p*<0.05) (Table 1).

All patients claimed to be religious. Most of the patients in the MDRS group had a family income of less than or equal to the minimum wage (*p*<0.05), whereas most of the patients in the other groups had a family income exceeding the minimum wage (Table 1). Most of the patients in the MDRS group and the MDWS group did not work (*p*<0.05), whereas most of those in the control group worked (*p*<0.05) (Table 1).

When health conditions were evaluated, it was clear that most of the sample did not report their number of hospitalizations (Table 2). When compared with the other groups (Table 2), the MDRS group had the highest number of patients who reported that they had relatives with mental disorders (*p*<0.05).

The MDWS group reported being more physically active than the other groups (*p*>0.05) (Table 2). The MDRS group reported more diseases (diabetes, hypertension and heart problems) (*p*>0.05) (Table 2).

Among the 202 patients in the MDRS group, 168 had already attempted suicide, but only 73 reported suicidal ideation in the last week. In this group, the risk of suicide attempts was mild, followed by severe and moderate risks. Among the 207 patients in the MDWS group, 42 had already attempted suicide, but only four reported suicidal ideation in the last week. None of the patients from the CO group presented suicide risks in the MINI 5.0 (Table 2).

In the general sample, it is clear that the 'environment' domain received the lowest score. In the MDRS group, the lowest-scoring domains were the 'environment', 'physical' and 'psychological'. In the MDWS and CO groups, the lowest-scoring domain was the 'environment'. Statistical correlation has been found between these two groups (*p*<0.05). Notably, the patients in the MDRS group presented the lowest quality of life compared with the other groups (Table 3).

The MINI 5.0 revealed that depressive disorders, psychotic syndromes, manic and hypomanic episodes, generalized anxiety disorder, social phobias, post-traumatic stress disorder and obsessive compulsive disorder are more common and statistically significant in the MDRS group (*p*<0.05) (Table 4).

## DISCUSSION

The average age in the three groups was 36.4 (MDRS), 39.2 (MDWS) and 37.0 years (CO). Some studies show that these averages are within the age range of patients who reported more suicide attempts in the southern region of Brazil (30 to 39 years of age) 10 and in Ceará (30 to 59 years of age) 11. A study conducted in Alagoas found that the average age of patients who reported suicide attempts was younger (20.1 years) 12, which conflicts with our study's findings.

Most of the interviewed patients were female, perhaps because the greatest demand for health services in Arapiraca comes from women. This gender ratio may present a bias in this study; however, because suicide attempts are more frequent among women 10,11,12,13, this ratio does not negate the findings of the study. Women attempt suicide more often than men, but men choose more lethal means to achieve that purpose 10.

One study found that a higher risk of suicide exists among single and divorced/widowed people compared with married people 14. Our data corroborate this finding. However, a study conducted in southern Brazil showed that married women attempted suicide more often than other women did 10.

We found that the patients in the MDRS group had a lower level of education than patients in the other groups did. A low educational level is common among individuals who attempt suicide 10,11,13. Researchers should consider whether a low level of education may be related to a previous mental disorder that reduces the individual's likelihood of achieving a higher level of education 15.

A rarely studied area involves several possible mechanisms by which religion can be associated with a lower risk of suicide 15. We found no difference between the groups with regard to religion. Isolation and permanent feelings of exclusion may cause people who experience difficulties that are rarely discussed to distance themselves from religion, which could provide them with possible solutions and relief from their suffering 16. A study conducted in Ceará showed that most patients who attempted suicide were religious (96.5%) 11.

Most of the patients in the MDRS group had lower family incomes than patients in the other groups. According to the WHO 17, risk factors for suicide are social extremes, including low and high incomes. Some studies show that individuals who have attempted suicide often belong to a low economic class 5,11, particularly identifying the classes C, D and E 13.

Most of the patients in the MDRS and MDWS groups did not work, whereas most of those in the CO group worked. Mental disorders might hinder patients in the MDRS and MDWS groups from entering the labor market. Some studies show that unemployment is a risk factor for suicide attempts 5,11. However, studies have found suicide attempts among some workers 10.

A study conducted in Ceará showed that 68% of patients who attempted suicide had a history of psychiatric hospitalization 11. In this study, it was not possible to identify how many times the patients were hospitalized to evaluate how the number of hospitalizations related to the risk of suicide attempts.

A family history of mental disorders increases the risk of suicidal behavior 2. In this study, we found that most patients in the MDRS group had relatives with mental disorders and that this was more common in the MDRS group than in other groups.

Some studies have shown that degenerative diseases contribute to suicidal behavior 18. A study in the Republic of Korea found that heart problems and diabetes mellitus were risk factors for suicide 19. However, a study in Australia did not identify diabetes mellitus as a risk factor for suicide 20. Our study found that patients in the MDRS group showed the highest frequency of chronic degenerative diseases, including diabetes, hypertension and heart problems.

In all three groups, we found that the 'environment' domain performed worse than the other domains. The patients in the MDRS group showed lower quality of life when compared to the other groups. In individual terms, emotional resiliency, the ability to solve problems and certain social skills can reduce the impact of adverse environmental factors or intrapsychic factors and can counterbalance the weight of some risk factors 2. Suicide is a complex phenomenon with various causes, and it is an important indicator of a population's quality of life 21.

The presence of a mental disorder is one of the most important risk factors for suicide. In general, it is believed that 90% to 98% of those who commit suicide have a mental disorder 2,22,23. Our study showed that depressive disorders, manic episodes, hypomanic episodes, social phobias, obsessive compulsive disorder, post-traumatic stress disorder, psychotic syndromes and generalized anxiety disorder are more common in patients in the MDRS group.

Mood disorders, particularly depressive disorders, represent the most frequent diagnoses among patients with mental disorders who committed suicide 2,24,25. In addition, studies show that the presence of depression 13,26 and anxiety disorders 13,27 increases the risk of suicide.

Depression was the most common mental disorder in the MDRS group. Despite the severity of depression and the availability of effective treatments, only 30% of cases worldwide receive the necessary care 28. Estimates show that by 2020, depression will be the second most common disease and will be more prevalent in all age groups of both sexes worldwide 28.

This study suggests that urgent mental health care measures should be implemented for patients with mental disorders who present a higher risk of suicide attempts. Otherwise, there will be a considerable increase in the number of suicide attempts and, in turn, in the number of suicides.

This study found that patients with mental disorders who had a risk of suicide attempts were mostly single, had a lower level of education, had a lower family income and did not work. They also experienced suicidal ideation in the previous week, had a lower quality of life and suffered from depressive disorders, manic episodes, hypomanic episodes, social phobias, obsessive compulsive disorder, post-traumatic stress disorder, psychotic syndromes and/or generalized anxiety disorder.

The management of patients with a risk of suicide must be revised to enable changes in the patients' quality of life and the care devoted to the mental disorders identified. A patient who does not receive the necessary care may be more likely to attempt suicide. Therefore, offering a mental health service network that facilitates patients' access to care may reduce or prevent suicide attempts and contribute to the improvement of these patients' quality of life.

The limitation of this study is related to the difficulty of interviewing men in health services in Arapiraca. A more homogeneous sample with regard to sex would make it possible to identify the profile of mental disorders, suicide attempts and quality of life in both sexes, although men do not usually attend this type of service.

## ACKNOWLEDGMENTS

The authors are grateful for the support of the National Council for Scientific and Technological Development.

## AUTHOR CONTRIBUTIONS

Alves VM designed the study, wrote the protocol, managed the literature searches, analyzed the data, wrote the manuscript and performed the statistical analyses. Lima Francisco LC, Pereira Belo FM, de-Melo-Neto VL and Barros VG analyzed the data and wrote the manuscript. Nardi AE contributed to a critical review of the paper. This research is part of a doctoral thesis in Psychiatry and Mental Health by Veronica de Medeiros Alves, Institute of Psychiatry, Federal University of Rio de Janeiro. All authors contributed to and approved the final manuscript.

## ACKNOWLEDGMENTS

The authors are grateful for the support of the National Council for Scientific and Technological Development.

## Figures and Tables

**Table 1:** General characteristics of the sample (n=605).

General data	MDRS*	MDWS*	CO*	
Age				t
Mean (SD)	36.4 (11.3)	39.2 (13.4)	37.0 (12.8)	0.001
Sex				
Female/Male	164/38	173/34	171/25	
Marital status				
Married	99 (49.0%)	137 (66.2%)	114 (58.2%)	
Single/Divorced	103 (51.0%)	70 (33.8%)	82 (41.8%)	
Schooling				
Complete and incomplete higher education	5 (2.5%)	23 (11.1%)	46 (23.5%)	
Complete and incomplete high school	40 (19.8%)	56 (27.1%)	67 (34.2%)	
Complete and incomplete elementary school	157 (77.7%)	128 (61.8%)	83 (42.3%)	
Religion				
Religious	189 (93.6%)	193 (93.2%)	186 (94.9%)	
Not religious	13 (6.4%)	14 (6.8%)	10 (5.1%)	
Family income				
Up to 1 minimum wage earner	119 (58.9%)	91 (43.9%)	69 (35.2%)	
More than 1 minimum wage earner	83 (41.1%)	116 (56.1%)	127 (64.8%)	
Employment				
Yes	49 (24.2%)	81 (39.1%)	105 (53.6%)	
No	153 (75.8%)	126 (60.9%)	91 (46.4%)	

**<?ENTCHAR ast?>:** MDRS: mental disorder patients and risk of suicide attempts; MDWS: mental disorder patients without risk of suicide attempts; CO: control.

**Table 2:** Characteristics of the health conditions of the sample (n=605).

Health conditions	MDRS*	MDWS*	CO*
Number of hospitalizations			
Up to 2	21 (10.4%)	7 (3.4%)	3 (1.5%)
More than 2	29 (14.4%)	12 (5.8%)	2 (1.0%)
None	152 (75.2%)	188 (90.8%)	191 (97.5%)
Relatives with mental disorders			
Yes	120 (59.4%)	89 (43.0%)	66 (33.7%)
No	72 (35.6%)	107 (51.7%)	107 (54.6%)
Do not know	10 (5.0%)	11 (5.3%)	23 (11.7%)
Physical activity			
Yes	41 (20.3%)	60 (29.0%)	52 (26.5%)
Comorbidity			
Diabetes	20 (9.9%)	15 (7.2%)	9 (4.6%)
Hypertension	37 (18.3%)	32 (15.5%)	25 (12.8%)
Heart problems	10 (5.0%)	6 (2.9%)	6 (3.1%)
Other	22 (10.9%)	30 (14.5%)	20 (10.2%)
Number of suicide attempts	168 (83.2%)	42 (20.3%)	0
1 time	67 (39.9%)	26 (61.9%)	0
2 to 3 times	28 (16.7%)	7 (16.7%)	0
More than 4 times	62 (36.9%)	5 (11.9%)	0
Not reported	11 (6.5%)	4 (9.5%)	0
Current suicidal ideation			
Yes	73 (36.1%)	4 (1.9%)	0
Risk of suicide (MINI)			
Mild	101 (50.0%)	0	0
Moderate	12 (5.9%)	0	0
Severe	89 (44.1%)	0	0

**<?ENTCHAR ast?>:** MDRS: mental disorder patients and risk of suicide attempts; MDWS: mental disorder patients without risk of suicide attempts; CO: control.

**Table 3:** Average and standard deviation of domains in relation to the presence of mental disorders and the risk of suicide attempts.

Mean (SD)	Physical	Psychological	Social relations	Environment	General quality of life	*p*
**General**	62.3 (17.8)	63.7 (17.4)	66.0 (17.6)	53.5 (14.4)	64.1 (20.7)	0.000
MDRS*	50.5 (17.4)	51.2 (18.3)	57.4 (18.3)	46.9 (14.7)	52.9 (22.4)	0.000
MDWS*	64.0 (15.4)	65.6 (13.3)	66.6 (15.9)	53.6 (12.4)	66.3 (17.8)	0.000
CO*	72.6 (12.6)	74.4 (11.2)	74.2 (12.4)	60.0 (12.9)	72.9 (16.2)	0.000

**<?ENTCHAR ast?>:** MDRS: mental disorder patients with risk of suicide attempts; MDWS: mental disorder patients without risk of suicide attempts; CO: control.

Significant difference between the MDRS, MDWS and CO groups (*p*=0.000) (two-way ANOVA).

Presented correlation between the MDWS and CO groups (*p*=0.001) (Pearson correlation coefficient).

**Table 4:** Frequency of mental disorders in patients with and without risk of suicide attempts.

Mental disorder	General*			MDRS*				MDWS*			*OR*	*p*
		n	%		n	%	n	%				
Current major depressive episode	209	34.5	139		68.8		70		33.8		4.3	**0.000**
Recurrent major depressive episode	148	25.3	89		44.1		59		28.5		2.0	**0.001**
MDE with melancholic features	139	23.0	98		48.5		41		19.8		3.8	**0.000**
Dysthymic disorder	30	5.0	22		10.9		8		2.9		3.3	**0.006**
Manic episode	133	22.0	84		41.6		49		23.7		2.6	**0.000**
Hypomanic episode	27	4.5	19		9.4		8		3.9		2.6	**0.041**
Panic disorder	53	8.8	31		15.3		22		10.6		1.7	0.123
Agoraphobia	184	30.4	93		46.0		91		44.0		1.1	0.747
Social phobia	69	11.4	50		24.8		19		9.2		3.2	**0.000**
Obsessive compulsive disorder	48	7.9	39		19.3		9		4.3		5.3	**0.000**
Post-traumatic stress disorder	52	8.6	40		19.8		12		5.8		4.0	**0.000**
Alcohol abuse/dependency	37	6.1	21		10.4		16		7.7		1.5	0.325
Substance abuse/dependency	12	2.0	7		3.5		5		2.4		1.5	0.650
Psychotic syndrome	162	26.8		106		52.5		56		27.0	3.6	**0.000**
Mood disorder with psychotic features	74	12.2		60		29.7		14		6.8	5.8	**0.000**
Anorexia nervosa	0	0		0		0		0		0	-	-
Bulimia nervosa	5	0.8		3		1.5		2		1.0	1.5	0.978
Generalized anxiety disorder	147	24.3		94		46.5		53		25.6	2.5	**0.000**
Antisocial personality disorder	6	1.0		4		2.0		2		1.0	2.1	0.659
Total	605	100.0		202		100.0		207		100.0		

**<?ENTCHAR ast?>:** General: all patients studied; MDRS: mental disorder patients with risk of suicide attempts; MDWS: mental disorder patients without risk of suicide attempts. Chi-square tests were used to compare the MDRS, TMRS and MDWS groups.
